# 1884. Evaluating SARS-CoV-2 Surveillance Strategies at the United States Naval Academy: A Comparison of Saliva and Dried Blood Spot Serosurveillance Against Molecular-Confirmed Case Detection

**DOI:** 10.1093/ofid/ofac492.1511

**Published:** 2022-12-15

**Authors:** Liana R Andronescu, Stephanie A Richard, Eric D Laing, Adam K Saperstein, Jitendrakumar Modi, Christopher D Heaney, Jamie A Fraser, Saira Shaikh, Christopher C Broder, Timothy H Burgess, Simon Pollett, Eugene V Millar, Christian L Coles, Mark P Simons

**Affiliations:** Henry M. Jackson Foundation for the Advancement of Military Medicine Inc., in support of the Infectious Disease Clinical Research Program, Silver Spring, Maryland; Infectious Disease Clinical Research Program, Department of Preventive Medicine and Biostatistics, Uniformed Services University of the Health Sciences, Bethesda, MD, USA, Bethesda, MD; Department of Microbiology and Immunology, Uniformed Services University, Bethesda, MD, Bethesda, Maryland; Department of Family Medicine, Uniformed Services University, Bethesda, MD, Annapolis, Maryland; Naval Health Clinic, Annapolis, MD, Anapolis, Maryland; Department of Environmental Health and Engineering, Bloomberg School of Public Health, Johns Hopkins University, Baltimore, Maryland; Infectious Disease Clinical Research Program, Department of Preventive Medicine and Biostatistics, Uniformed Services University of the Health Sciences, Bethesda, MD, USA, Bethesda, MD; Infectious Disease Clinical Research Program, Department of Preventive Medicine and Biostatistics, Uniformed Services University of the Health Sciences, Bethesda, MD, USA, Bethesda, MD; Department of Microbiology and Immunology, Uniformed Services University, Bethesda, MD, Bethesda, Maryland; Infectious Disease Clinical Research Program, Department of Preventive Medicine and Biostatistics, Uniformed Services University, Bethesda, MD, Bethesda, Maryland; Infectious Disease Clinical Research Program, Department of Preventive Medicine and Biostatistics, Uniformed Services University of the Health Sciences, Bethesda, MD, USA, Bethesda, MD; Infectious Disease Clinical Research Program, Department of Preventive Medicine and Biostatistics, Uniformed Services University, Bethesda, MD, Bethesda, Maryland; Infectious Disease Clinical Research Program, Department of Preventive Medicine and Biostatistics, Uniformed Services University of the Health Sciences, Bethesda, MD, USA, Bethesda, MD; Infectious Disease Clinical Research Program, Department of Preventive Medicine and Biostatistics, Uniformed Services University of the Health Sciences, Bethesda, MD, USA, Bethesda, MD

## Abstract

**Background:**

Congregate military populations remain at risk of SARS-CoV-2 outbreaks and the optimal surveillance approach in such settings remains unclear. We enrolled midshipmen at the United States Naval Academy (USNA) in a setting of frequent PCR screening use of prevention strategies.

**Methods:**

Dried blood spots (DBS) and saliva were collected in August 2020, December 2020, February 2021 (saliva only) and April/May 2021 to measure anti-SARS-CoV-2 spike (S) and nucleoprotein (NP) IgG. COVID-19 vaccine history and records of SARS-CoV-2 PCR tests and routine asymptomatic screening assays were obtained from the USNA Brigade Medical Clinic. Attack rates were compared with cumulative frequencies of infections. Concordance of saliva and DBS anti-NP and anti-S IgG positivity was determined using Cohen’s kappa coefficient.

**Results:**

The study enrolled 181 midshipmen. COVID-19 vaccinations were administered in March/April 2021. Samples were collected for 101 participants in August, 73 in December, 57 in February (saliva only), and 63 in April/May. In August, 17 (17%) participants showed evidence of SARS-CoV-2 infection based on anti-S IgG values from DBS and/or saliva. By December 2020, anti-S seroconversion was observed for 5 more based on DBS and/or saliva. By May 2021, 100% of participants were anti-S IgG seropositive after vaccination based on DBS and/or saliva; 48% of participants had seroconverted to anti-NP IgG. Among participants with both DBS and saliva samples, a coefficient of 0.64 showed substantial agreement between anti-S IgG results in August and perfect agreement in December (Table 1). DBS and saliva results for anti-NP IgG were in perfect agreement through December and in substantial agreement in May (0.68, Table 2). Prior to vaccination in March/April 2021, 4/48 of participants had at least one documented SARS-CoV-2 PCR positive result (Table 3). Cumulative PCR test positivity concordance with DBS seroconversion was 37.5% and 60% for anti-S IgG and anti-NP IgG, respectively.

**Figure:**
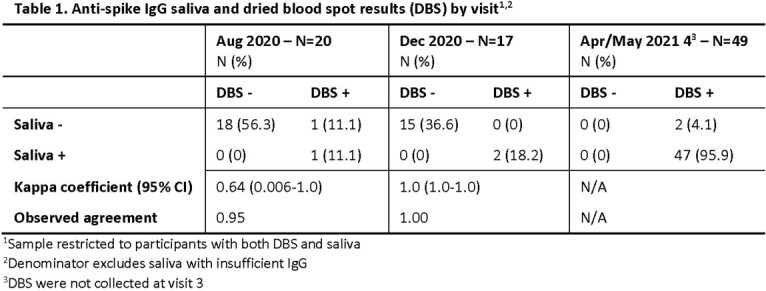

**Figure:**
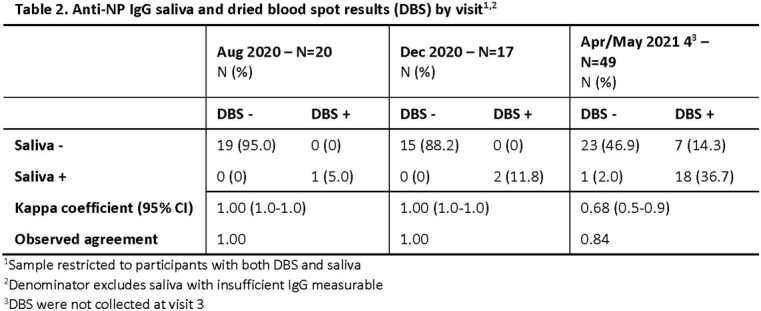

**Figure:**
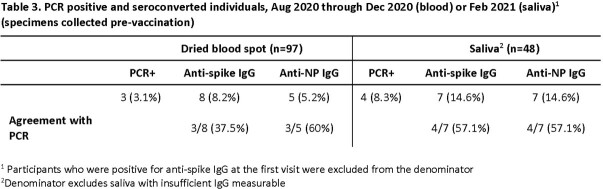

**Conclusion:**

There was a substantive SARS-CoV-2 attack rate before vaccination; all vaccinees mounted an anti-S IgG response in blood. We note high agreement between DBS and saliva for IgG measurement. Serology-based surveillance identified substantially more SARS-CoV-2 infections than PCR screening.

**Figure:**
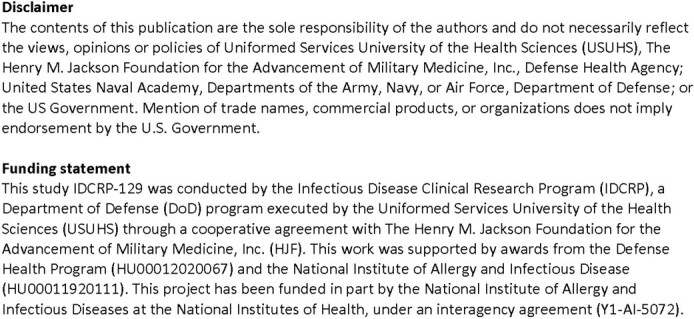

**Disclosures:**

**Jitendrakumar Modi, MD**, GlaxoSmithKline: I am a paid speaker for GSK. I do not speak for their flu brand. **Timothy H. Burgess, MD, MPH**, AstraZeneca: The HJF, in support of the USU IDCRP, was funded to conduct or augment unrelated Phase III Mab and vaccine trials as part of US Govt. COVID19 response **Simon Pollett, MBBS**, Astra Zeneca: The HJF, in support of the USU IDCRP, was funded to conduct or augment unrelated Phase III Mab and vaccine trials as part of US Govt. COVID19 response **Mark P. Simons, PhD**, AstraZeneca: The HJF, in support of the USU IDCRP, was funded to conduct or augment unrelated Phase III Mab and vaccine trials as part of US Govt. COVID19 response.

